# Nutritional support in pancreatic cancer patients and its effect on nutritional status: an observational regional HPB network study investigating current practice

**DOI:** 10.1007/s00520-024-08683-0

**Published:** 2024-07-05

**Authors:** Allard G. Wijma, Rianne N. M. Hogenbirk, Heleen Driessens, Daniëlle A. Kluifhooft, Ellen S. Jellema-Betten, Marlies Tjalsma-de Vries, Mike S. L. Liem, Vincent B. Nieuwenhuijs, Eric M. Manusama, Frederik J. H. Hoogwater, Maarten W. Nijkamp, Sandra Beijer, Joost M. Klaase

**Affiliations:** 1 Department of Surgery, Division of Hepato-Pancreato-Biliary Surgery and Liver Transplantation, University Medical Center Groningen, University of Groningen, Groningen, The Netherlands; 2 Department of Surgery, Isala Clinics, Zwolle, The Netherlands; 3 Department of Dietetics and Nutrition, University Medical Center Groningen, University of Groningen, Groningen, The Netherlands; 4 Department of Surgery, Medical Spectrum Twente, Enschede, The Netherlands; 5 Department of Surgery, Medical Center Leeuwarden, Leeuwarden, The Netherlands; 6 Department of Research & Development, Netherlands Comprehensive Cancer Organisation (IKNL), Utrecht, The Netherlands

**Keywords:** Pancreatic cancer, Dietary supplements, Malnutrition, Prehabilitation, Surgical outcomes

## Abstract

**Purpose:**

Preoperative malnutrition is associated with poor postoperative outcomes in patients with pancreatic cancer. This study evaluated the effectiveness of current practice in nutritional support for patients with pancreatic cancer.

**Methods:**

Observational multicenter HPB network study conducted at the Isala Clinics Zwolle, Medical Spectrum Twente, Medical Center Leeuwarden, and University Medical Center Groningen between October 2021 and May 2023. Patients with a suspected pancreatic malignancy scheduled for surgery were screened for malnutrition using the Patient-Generated Subjective Global Assessment (PG-SGA) questionnaire and referred to a dedicated dietician for nutritional support comprising pancreatic enzyme replacement therapy, dietary advice, and nutritional supplements to achieve adequate caloric and protein intake. At baseline, 1 day preoperatively, and 3 months postoperatively, the nutritional status and muscle thickness were evaluated.

**Results:**

The study included 30 patients, of whom 12 (40%) classified as malnourished (PG-SGA ≥ 4) at baseline. Compared to well-nourished patients, malnourished patients were younger, were predominantly female, and had a higher body mass index, despite having lost more body weight in the past 6 months. All malnourished patients and 78% of the well-nourished patients received nutritional support. Consequently, a preoperative increase in caloric and protein intake and body weight were observed. Postoperatively, despite a further increase in caloric intake, a considerable decrease in protein intake, body weight, and muscle thickness was observed.

**Conclusion:**

Malnutrition is prevalent in patients undergoing pancreatic surgery. Nutritional support by a dedicated dietician is effective in enhancing patients’ preoperative nutritional status. However, postoperative monitoring of adequate nutritional intake in patients could be improved.

**Supplementary Information:**

The online version contains supplementary material available at 10.1007/s00520-024-08683-0.

## Introduction

Pancreatic cancer is an aggressive malignancy, and surgical resection remains the most important treatment modality to provide patients with the best chance of long-term survival [1]. However, pancreatic surgery is highly invasive, and patients’ postoperative recovery is often impeded by (severe) postoperative complications [2]. Currently, greater attention is paid to the preoperative optimization of patient-related modifiable risk factors, a practice known as prehabilitation, with the aim of enhancing postoperative outcomes in patients. Multiple patient-related risk factors are identified as valid indicators for poor postoperative outcomes and are deemed modifiable in the preoperative phase in patients undergoing major abdominal surgery [3, 4]. One significant preoperative risk factor in patients with pancreatic cancer is the development and progression of malnutrition, a complex condition characterized by increased tumor metabolism, inadequate nutrient intake, and malabsorption [5]. Severe malnutrition leads to cachexia, a metabolic syndrome characterized by the pathological loss of skeletal muscle mass and adipose tissue [6]. Ultimately, malnutrition contributes to poor surgical and oncological outcomes due to a reduced physical reserve in patients which is necessary to withstand the physical demands of surgery [7–11].

Malnutrition is frequently observed in patients with cancer, with incidence rates ranging from 50 to 80% being reported [7, 12–14]. The multifactorial etiology of malnutrition in patients with pancreatic cancer encompasses various factors, including tumor-related factors, pancreatic endocrine and exocrine insufficiency, disease-related symptoms, and treatment-related side effects [5, 15, 16]. In particular, the endocrine and exocrine function of the pancreas can be affected in patients with pancreatic cancer. Endocrine pancreatic insufficiency can lead to type 3c diabetes mellitus (DM), also known as pancreatogenic DM, and contributes to maldigestion and malabsorption of nutrients [5]. However, exocrine pancreatic insufficiency, in particular, contributes to the malabsorption of essential nutrients due to the inadequate secretion of digestive enzymes into the small intestine [5]. Treatment for exocrine pancreatic insufficiency typically involves pancreatic enzyme replacement therapy (PERT), which includes taking digestive enzymes with meals to aid in digestion and absorption [9, 17]. Additionally, dietary modifications and, if necessary, oral nutritional supplements or tube feeding may be recommended in order to address caloric and protein deficiencies [9]. Notably, only a few years ago, Latenstein et al. found that nearly half of the malnourished patients with pancreatic cancer did not receive preoperative nutritional support [18].

In the current study, we sought to determine the effectiveness of current practice concerning preoperative nutritional support in (malnourished) patients with pancreatic cancer undergoing surgery in a regional hepato-pancreato-biliary (HPB) network.

## Material and methods

### Study design

This observational multicenter study was conducted between October 2021 and May 2023 in a regional HPB network consisting of four hospitals: Isala Clinics Zwolle, Medical Spectrum Twente, Medical Center Leeuwarden, and University Medical Center Groningen (UMCG). The aim of this study was to investigate the current practice concerning nutritional support in patients undergoing pancreatic surgery. Hereto, in all consecutive patients over 18 years old with a suspected pancreatic malignancy who were scheduled for an elective pancreatoduodenectomy and had provided informed consent, it was recorded whether nutritional support was provided perioperatively and what this support consisted of. Patients requiring neoadjuvant chemotherapy were excluded. All included patients were screened for malnutrition using the Patient-Generated Subjective Global Assessment Short Form (PG-SGA SF) questionnaire and were classified as either malnourished (score ≥ 4) or well nourished (score < 4) [19]. Additionally, malnourished patients were compared to well-nourished patients and the impact of perioperatively provided nutritional support was evaluated. This study was approved by the Medical Ethics Committee of the UMCG (Netherlands research register number 202000299), and written consent was obtained from all patients prior to inclusion. The study was performed in accordance with the ethical standards as stated in the 1964 Declaration of Helsinki and its later amendments. Lastly, STROBE guidelines were adhered to as applicable for this study [20].

### Perioperative nutritional support: current practice

Whether patients were preoperatively referred to a dedicated dietician for nutritional support was at the discretion of the consulting surgeon. In all participating hospitals, a dedicated dietician conducted a comprehensive nutritional assessment for patients who were referred for nutritional support to determine their specific dietary requirements [21]. Based on this assessment, the dietician gave patients dietary advice to achieve adequate caloric and protein intake and provided them with a prescription for nutritional supplements, tube feeding, or parenteral nutrition if necessary. Furthermore, the dietician initiated PERT if patients had symptoms of exocrine pancreatic insufficiency. Lastly, the dietician continuously evaluated the nutritional treatment plan, with active and regular follow-up of the patient.

### Study objectives

The primary objective of this study was to assess the perioperative referral rates for nutritional support among patients suspected of pancreatic cancer and scheduled to undergo pancreatic surgery. Also, it was recorded what the nutritional support consisted of. Additionally, the study aimed to evaluate the efficacy of optimizing the nutritional status, particularly in malnourished patients, during the relatively brief preoperative period.

### Study assessments

To evaluate the effects of nutritional support on patients’ nutritional status, nutritional intake, muscle thickness, and functional capacity in both the preoperative and postoperative periods, the below mentioned study assessments were performed at inclusion within a week of the patients’ visit to the surgical outpatient department (T0), 1 day prior to surgery (T1), and 3 months after surgery (T2).

#### Nutritional status and support

To assess their nutritional status, patients were asked to fill out the Patient-Generated Subjective Global Assessment Short Form (PG-SGA SF) questionnaire [19]. The questionnaire is patient-led and evaluates alterations in body weight, dietary intake, gastrointestinal symptoms, and functional capacity during the past month. The questionnaire has been found to effectively identify malnutrition in patients with cancer [22]. Subsequently, during each study visit, body mass index (BMI) was calculated. Additionally, the total number of patients preoperatively referred to a dedicated dietician was recorded. Regarding referred patients, the total number of preoperative consultations (either face-to-face or by telephone) was registered. Furthermore, the number of days between the first consultation with the dietician and the day of surgery was listed. Patients’ utilization of oral nutritional supplements, tube feeding, or parenteral nutrition to complement their dietary intake was documented. Lastly, whether and when patients started PERT was recorded.

#### Nutritional intake

Patients were asked to keep a nutritional diary and to record their dietary intake for three consecutive days prior to each study visit. Based on these nutritional diaries, energy intake and protein intake were calculated using an online nutrition calculator (Mijn Eetmeter, Stichting Voedingscentrum Nederland, Rotterdam, the Netherlands). Energy intake was expressed in kilocalories (kcal) per kilogram (kg) of body weight per day, whereas protein intake was expressed in grams (g) of protein per kilogram of body weight per day.

#### Muscle thickness

**Fig. 1:**
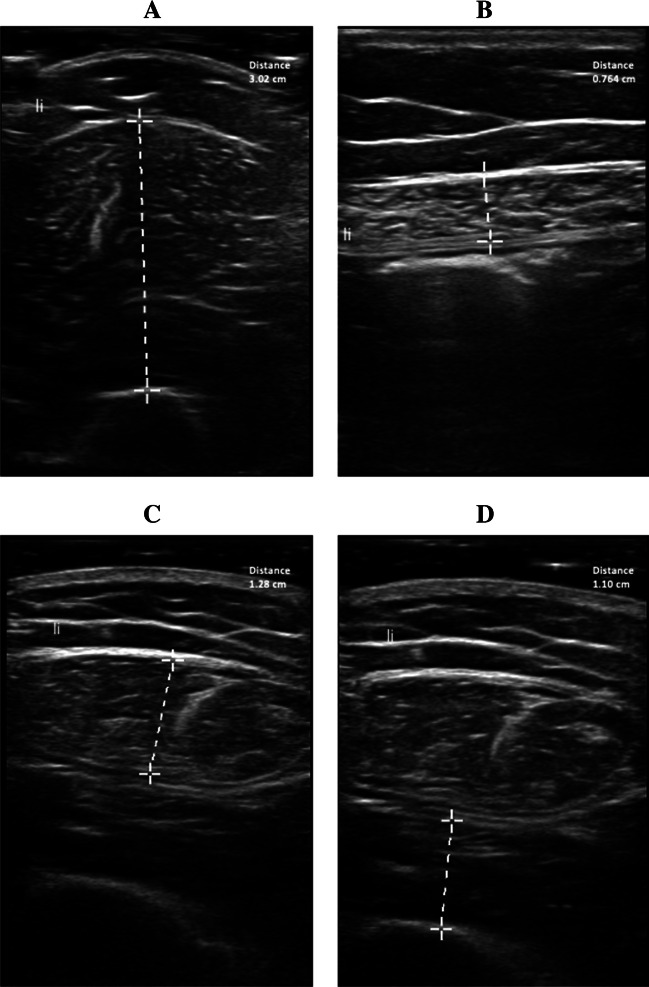
Representative images of the ultrasound muscle thickness measurements of the **A** m. biceps brachii, **B** m. rectus abdominis, **C** m. rectus femoris, and **D** m. vastus intermedius

Previous research indicated that combined muscle thickness measurement performed by point-of-care ultrasound (POCUS) is a valid indicator for skeletal muscle status [23]. Therefore, muscle thickness of the m. biceps brachii, m. rectus femoris, m. vastus intermedius, and m. rectus abdominis was measured in patients by three researchers (R.N.M.H., A.G.W., and D.K.) using POCUS (Philips FUS6882 Lumify L12-4, Koninklijke Philips N.V., the Netherlands) according to a previously published protocol [24]. The average of these measurements was used for the final analysis. An example of the acquired images is provided in Fig. 1.

#### Functional capacity

Patients’ functional capacity was assessed by performing the five times sit to stand test (FTSST). The FTSST is a validated test used to assess functional independence in patients; it includes the assessment of lower limb strength, balance control, and mobility [25]. Moreover, as a proxy for muscle strength, patients’ handgrip strength was assessed using a handheld dynamometer (Jamar FAB12- 0604 + , JLW Instruments, Chicago, IL, USA) [26].

#### Patient characteristics and postoperative surgical outcomes

Baseline characteristics (i.e., age, sex, American Society of Anesthesiology (ASA) score, smoking status, and relevant comorbidities), intraoperative variables (i.e., operation time and estimated blood loss), and 30-day postoperative outcomes (i.e., length of hospital stay, complications graded according to the Clavien-Dindo classification [27], length of intensive care unit (ICU) or medium care unit (MCU) stay, ICU and MCU readmission rate, unplanned hospital readmission, and 30-day mortality rate) were collected and analyzed.

### Statistical analysis

In light of the observational and explorative design of this study, no formal statistical tests were executed. For both patient characteristics and study-specific outcomes between malnourished patients and well-nourished patients, descriptive statistics were applied. For all continuous variables, the median and interquartile range are provided to convey information about the distribution of variables among the study cohort. Categorical data are presented as numbers and percentages. Paired line graphs were used to represent the distribution of daily energy intake, daily protein intake, body weight, and muscle thickness measurements during each study visit for malnourished and well-nourished patients. Additionally, the daily energy intake and protein intake of individual malnourished patients were represented in parallel plots. The R software version 4.2.2. (R Foundation for Statistical Computing, Vienna, Austria) was used for descriptive analyses, and GraphPad Prism version 10.0.2 (GraphPad Software, Boston, Massachusetts, USA) was used to represent the data.

## Results

### Study cohort

A total of 30 patients were included in this study. In Table 1, the baseline characteristics of the included patients are provided. Twelve patients (40%) were classified as malnourished, and 18 (60%) were classified as well-nourished, based on preoperative PG-SGA SF scores. Malnourished patients were overall younger (60.5 years versus 70 years, respectively), were predominantly female (75% versus 44%, respectively), and had a higher BMI (26.0 kg/m^2^ versus 23.5 kg/m^2^, respectively), despite having lost more body weight in the past 6 months, compared to well-nourished patients.

**Table 1:** Baseline characteristics

	Malnourished, *n* = 12 (40%)	Well-nourished, *n* = 18 (60%)
Age (years)	60.5 (58.0–71.3)	70 (59.8–74.5)
Female sex (*n* (%))	9 (75)	8 (44)
Body mass index (kg/m^2^)	26.0 (22.4–31.3)	23.5 (22.2–24.8)
Preoperative body weight loss in past 6 months (*n* (%))		
≤ 5% of body weight	4 (33)	10 (55)
5–10% of body weight	5 (42)	7 (39)
≥ 10% of body weight	3 (25)	1 (6)
ASA score ≥ 3 (*n* (%))	6 (50)	5 (28)
Comorbidities (*n* (%))		
Any comorbidity	10 (83)	12 (66)
Cardiac	2 (17)	3 (17)
Pulmonary	2 (17)	3 (17)
Renal	-	2 (11)
Diabetes mellitus	3 (25)	4 (22)
Hypertension	5 (42)	5 (28)
Smoking (*n* (%))		
Currently smoking	4 (33)	2 (11)
Stopped smoking	4 (33)	11 (61)
Never smoked	4 (33)	5 (28)
History of abdominal surgery (*n* (%))	6 (50)	6 (33)

Data are presented as a median (IQR) or number (%)

*PG-SGA SF* Patient-Generated Subjective Global Assessment short form, *ASA* American Society of Anesthesiologists

### Perioperative nutritional support and its effect on nutritional intake and body weight

In contrast to the 14 (78%) well-nourished patients, all 12 malnourished patients were referred to a dedicated dietician for perioperative nutritional support. The median time between the first consultation with the dietician and the day of surgery was 22 (14.5–38) days versus 25 (16–31) days for the malnourished and well-nourished patient cohorts, respectively. For patients in the malnourished group, an average of 1.8 follow-up consultations with the dietician were held preoperatively, whereas for patients in the well-nourished group, 1.4 follow-up consultations were held. The type of perioperative nutritional support prescribed to patients in both groups is provided in Table 2. Malnourished patients often required preoperative oral nutritional supplements to increase their caloric and protein intake. Furthermore, they regularly required preoperative PERT (75%) to correct for exocrine pancreatic insufficiency. Additionally, PERT was postoperatively prescribed to nearly all patients in the study cohort.

**Table 2:** Perioperative nutritional support

	Malnourished, *n* = 12 (40%)	Well-nourished, *n* = 18 (60%)
Preoperatively		
Oral nutritional supplements (*n* (%))	8 (67)	10 (56)
Enteral nutrition (*n* (%))	2 (17)	-
Parenteral nutrition (*n* (%))	-	-
PERT (*n* (%))	9 (75)	6 (33)
3 months postoperatively		
Oral nutritional supplements (*n* (%))	8 (67)	8 (44)
Enteral or parenteral nutrition (*n* (%))	-	-
PERT (*n* (%))	11 (92)	17 (94)

Data are presented as numbers (%)

*PERT* pancreatic enzyme replacement therapy

**Fig. 2:**
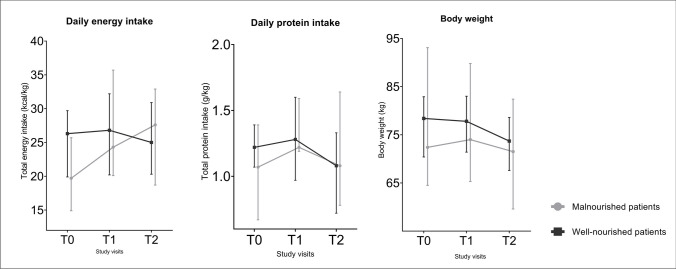
Nutritional intake and body weight over the course of the study period

As a result of the dietary advice and prescribed nutritional supplements, malnourished patients were able to increase their daily total energy intake from 19.7 (14.9–25.7) kcal/kg body weight/day at T0 to 24.3 (20.1–35.7) kcal/kg body weight/day at T1 and their daily total protein intake from 1.07 (0.67–1.39) g/kg body weight/day at T0 to 1.22 (1.19–1.59) g/kg body weight/day at T1. Although malnourished patients increased their daily total postoperative energy intake even more to 27.6 (18.7–32.9) kcal/kg body weight/day at T2, their daily total protein intake decreased to 1.08 (0.78–1.64) g/kg body weight/day at T2. Preoperatively, the median body weight increased from 72.4 (64.5–93.1) kg to 74.0 (65.3–89.8) kg for malnourished patients. However, postoperatively, the median body weight decreased to 71.5 (59.6–82.4) kg. For well-nourished patients, the daily total energy intake increased from 26.3 (19.9–29.7) kcal/kg body weight/day at T0 to 26.8 (20.2–32.2) at T1 and decreased to 25.0 (20.3–30.9) kcal/kg body weight/day at T2. Their daily total protein intake increased from 1.22 (1.07–1.39) g/kg body weight/day at T0 to 1.28 (0.97–1.60) g/kg body weight/day at T1 and decreased to 1.08 (0.72–1.33) g/kg body weight/day at T2. For the median body weight in well-nourished patients, a constant decline was observed from 78.4 (70.4–82.9) kg at T0 to 77.8 (71.4–83.0) kg at T1 and 73.7 (67.6–78.6) kg at T2. The dynamic changes in nutritional intake and body weight are graphically represented in Fig. 2.

Additionally, in the supplementals (Fig. S1), the individual changes in daily total energy and protein intake in malnourished patients are represented.

### Perioperative functional capacity and muscle thickness

An overview of the dynamic changes in perioperative functional capacity is provided in Table 3. Overall, the median handgrip strength increased slightly or remained relatively stable preoperatively but decreased postoperatively in both groups. Regarding the FTSST, no clinically relevant changes were observed in either group.

**Table 3:** Perioperative functional capacity

	Malnourished, *n* = 12 (40%)	Well-nourished, *n* = 18 (60%)
Baseline (T0)		
Handgrip strength (kg)		
Left	22.3 (17.1–30.3)	28.5 (20.4–32.1)
Right	23.8 (19.6–31.7)	30.5 (20.7–34.8)
5 times sit to stand test (seconds)	13.0 (11.6–16.2)	9.5 (8.3–10.5)
1 day preoperatively (T1)		
Handgrip strength (kg)		
Left	25.6 (17.2–30.2)	29.7 (21.0–34.3)
Right	25.5 (21.4–30.9)	27.7 (23.0–34.3)
5 times sit to stand test (seconds)	13.5 (10.1–14.7)	8.3 (7.3–10.4)
3 months postoperatively (T2)		
Handgrip strength (kg)		
Left	20.0 (14.9–35.3)	27.4 (20.4–34.6)
Right	23.1 (17.4–31.1)	26.8 (21.1–36.1)
5 times sit to stand test (seconds)	12.4 (10.5–16.1)	9.0 (8.0–11.9)

Data are presented as a median (IQR)

*kg* kilograms

**Fig. 3:**
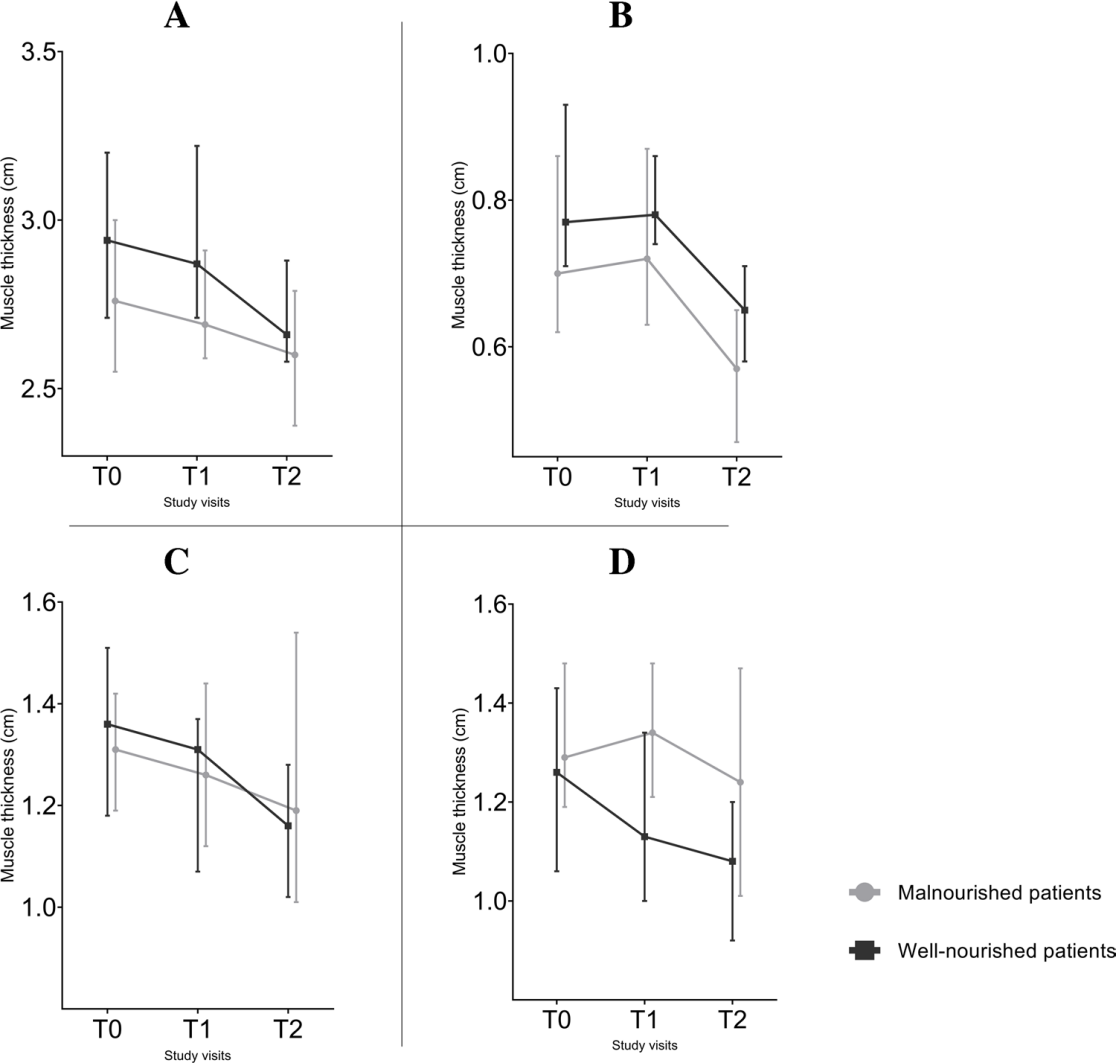
Muscle thickness of the **A** m. biceps brachii, **B** m. rectus abdominis, **C** m. rectus femoris, and **D** m. vastus intermedius

In Fig. 3, the dynamic changes in muscle thickness of the four muscle groups are graphically represented. The median muscle thickness decreased postoperatively for all muscle groups in both patient cohorts. However, postoperatively, the decrease in median muscle thickness was most prominent in patients who were preoperatively classified as well nourished.

### Surgical details

Finally, no noteworthy differences in surgical details were observed (Table 4). Postoperatively, the median length of stay was shorter for malnourished patients (9.5 days versus 12 days for well-nourished patients), and there were no unplanned readmissions for malnourished patients compared to 4 (22%) unplanned readmissions for well-nourished patients. Pancreatic adenocarcinoma was more frequently diagnosed in malnourished patients (75% versus 28% in well-nourished patients) and, consequently, they were more frequently treated with adjuvant chemotherapy (58% versus 11% in well-nourished patients).

**Table 4:** Surgical details

	Malnourished, *n* = 12 (40%)	Well-nourished, *n* = 18 (60%)
Surgical procedure (*n* (%))		
PPPD/PRPD	10 (83)	14 (78)
Whipple	1 (8)	3 (17)
Distal pancreatectomy	1 (8)	1 (6)
Duration of surgery (minutes)	393 (364–524)	402 (291–436)
Estimated intraoperative blood loss (mL)	375 (300–525)	350 (250–500)
R-status (*n* (%))		
R0	5 (42)	13 (72)
R1	7 (58)	5 (28)
Postoperative stay		
Length of ICU/MCU stay (days)	1 (1–1.3)	1 (0–1)
Length of hospital stay (days)	9.5 (8.8–12.3)	12 (7–18.8)
Complications		
Clavien-Dindo ≥ 3 (*n* (%))	3 (25)	4 (22)
Surgical reintervention (*n* (%))	3 (25)	2 (11)
In-hospital mortality (*n* (%))	-	-
Unplanned readmission < 30 days (*n* (%))	-	4 (22)
Histological diagnosis (*n* (%))		
Adenocarcinoma pancreas	9 (75)	5 (28)
Adenocarcinoma periampullary	1 (8)	6 (33)
IPMN	-	3 (17)
Neuroendocrine tumor	1 (8)	-
Other malignancy	-	3 (17)
No malignancy	1 (8)	1 (6)
Adjuvant chemotherapy*	7 (78)	2 (40)

Data are presented as a median (IQR) or a number (%)

*IPMN* intraductal papillary mucinous neoplasm. *PPPD* pylorus-preserving pancreatoduodenectomy, *PRPD* pylorus-resecting pancreatoduodenectomy, *mL* milliliters, *ICU* intensive care unit, *MCU* medium care unit

*In the Netherlands, adjuvant chemotherapy is solely prescribed to patients diagnosed with pancreatic adenocarcinoma. Hence, in this case, the percentage is in relation to adenocarcinoma pancreas

## Discussion

In this prospective observational multicenter study within a regional HPB network, we observed a high incidence of preoperative malnutrition in patients suspected of pancreatic cancer undergoing pancreatic surgery. The majority of malnourished patients were diagnosed with pancreatic adenocarcinoma. All malnourished patients and the majority of well-nourished patients were referred to a dedicated dietician for perioperative nutritional support. Furthermore, PERT and oral nutritional supplements were frequently prescribed to address exocrine pancreatic insufficiency and nutritional deficiencies, respectively. Notably, the implementation of the PACAP-1 trial in the Netherlands contributed to the current practice of referring numerous patients preoperatively to a dietician and prescribing PERT [28]. Nutritional support had a clear effect, with a preoperative increase in caloric and protein intake and body weight, and functional capacity remaining stable. However, despite a further increase in caloric intake, protein intake and body weight decreased substantially postoperatively. Moreover, across the entire study cohort, a considerable decline in muscle thickness was observed for the repeated measures of all four muscle groups.

The high incidence of malnutrition reported in this study aligns with previous research indicating that patients suspected of pancreatic cancer undergoing pancreatic surgery have an increased risk of preoperative malnutrition [7, 12–14]. If left untreated, malnutrition is associated with unfavorable surgical and oncological outcomes [11, 29, 30]. Therefore, the European Society for Clinical Nutrition and Metabolism (ESPEN) guidelines on nutrition in patients with cancer state that nutritional support must aim to mitigate metabolic derangements by optimizing nutritional intake to match the total energy expenditure of 25–30 kcal/kg/day and protein requirements of 1.2–1.5 g/kg/day in patients with cancer [31]. In this study, nutritional support improved preoperative energy intake in malnourished patients from 19.7 to 24.3 kcal/kg body weight/day and protein intake from 1.07 to 1.22 g/kg body weight/day. Well-nourished patients were also able to achieve adequate preoperative nutritional intake, which remained relatively stable (energy intake ranging from 26.3 to 26.8 kcal/kg body weight/day and protein intake ranging from 1.22 to 1.28 g/kg body weight/day). By comparison, the majority of previous studies investigating nutritional support in patients undergoing pancreatic surgery have solely focused on in-hospital postoperative nutritional intake [32, 33]. However, in a similar study, Min Park et al. reported that malnourished patients undergoing pancreatic surgery were provided with preoperative nutritional support and consequently achieved a high median caloric intake of 32.1 kcal/kg body weigh/day and a protein intake of 1.30 g/kg body weight/day [34]. Nonetheless, they commenced preoperative nutritional support only 1 week before surgery, casting doubt on its clinical relevance. Preferably, the time between the initiation of nutritional support and the surgery should be at least 14 days [35]. The latest ESPEN guidelines on nutrition in surgical patients highlight the importance of the timely commencement of preoperative nutritional support in malnourished patients and the delay of surgery, if necessary, to optimize nutritional status [36].

Adequate nutritional intake remains equally important in the postoperative phase to promote postoperative functional recovery. In this study, the postoperative total energy intake remained adequate, with an intake of 27.6 kcal/kg/day and 25.0 kcal/kg/day in the malnourished and well-nourished patient groups, respectively. However, in both groups, the total protein intake decreased to 1.08 g/kg/day, which might be due to fewer patients receiving oral nutritional supplements postoperatively. Maintaining an adequate nutritional status during adjuvant chemotherapy is crucial, because malnutrition has been linked to diminished tolerance to chemotherapy, increased treatment toxicity, lower treatment adherence, and the necessity for dose adjustments [37–39]. Conversely, the adverse effects of chemotherapy can also lead to inadequate nutrient absorption and intake and subsequent malnutrition [40]. The latter might also have influenced the results of this study, because more than half of the patients in the malnourished group received adjuvant chemotherapy during the T2 study visit.

In both study groups, a substantial postoperative decrease in body weight and muscle thickness was observed. The role of surgery-induced trauma in triggering the catabolic response, leading to a loss of muscle mass in the postoperative period, is widely recognized [41]. However, another explanation could be the decrease in postoperative protein intake. Insufficient nutritional intake in combination with insufficient physical activity was previously identified as the leading risk factor for postoperative loss of muscle mass [42]. Importantly, inadequate postoperative nutritional intake was previously associated with a decreased 1-year survival in patients undergoing cancer surgery [42].

The administration of chemotherapy is linked to muscle wasting, and the fact that several patients in this study received adjuvant chemotherapy might have adversely affected postoperative muscle thickness [43]. This once more highlights the importance of adequate postoperative nutritional support. Notably, the decrease in body weight and muscle thickness was most prominent in patients who were preoperatively classified as well-nourished. Previously, Hogenbirk et al. observed a similar phenomenon when investigating the occurrence of surgery-related muscle loss and its association with in-hospital nutritional intake in the first postoperative week in patients undergoing pancreatic surgery [44]. The authors suggested that this could be attributed to the malnourished patients’ inability to further lose muscle mass postoperatively. Additionally, having received extensive nutritional advice and support compared to well-nourished patients, malnourished patients might be more aware of the importance of sufficient nutritional intake following surgery.

The strengths of the present study include the implementation of a comprehensive and multifaceted approach to adequately assess the perioperative nutritional status of patients. Additionally, the multicenter design of this study provides a realistic overview of the management of malnutrition in patients suspected of pancreatic cancer undergoing surgery. Highlighting the significance of equal treatment for malnutrition within a regional HPB network is crucial, as practice variation is undesirable and may lead to suboptimal treatment of malnutrition. Nevertheless, a few limitations must be addressed. Firstly, the study’s explorative design with a small sample size increased the risk of a selection bias. Secondly, due to factors such as the severity of disease symptoms and preexisting conditions, considerable baseline variability was observed among included patients in nutritional intake, body weight, functional capacity, and muscle thickness. This variability might have resulted in regression to the mean, distorting true values, for example, in patients with particularly low muscle thickness. In future research, solely focusing on malnourished patients might be worthwhile. Finally, because this study had an observational design, the results could not be compared to a valid control group with malnourished patients who did not receive perioperative nutritional support. However, this study has provided novel insights into the effect of a systematic approach to nutritional support by a dedicated dietician on patients diagnosed with resectable pancreatic cancer.

In conclusion, preoperative nutritional support by a dedicated dietician improves the nutritional status of both malnourished and well-nourished patients. Therefore, we recommend, based on the findings of this study, that all patients undergoing pancreatic surgery be referred to a dedicated dietitian for a full nutritional assessment and subsequent preoperative nutritional support. Nevertheless, we simultaneously suggest that greater attention should be directed toward postoperative monitoring of adequate nutritional intake in patients.

### Supplementary Information

Below is the link to the electronic supplementary material.

Supplementary file1 (TIF 3650 KB) Figure S1. Individual malnourished patients’ daily total energy and protein intake during each study visit.

## Data Availability

The datasets generated or analyzed in the present study are not publicly available because the data are linked to a vulnerable patient population. However, these data are available upon reasonable request from the corresponding author (a.g.wijma@umcg.nl).
